# Supplementary material for: Comparison between Cardiac Output and Pulmonary Vascular Pressure Measured by Indirect Fick and Thermodilution Methods
Source: J Pers Med. 2023 Mar 20;13(3):559. doi: 10.3390/jpm13030559 (PMC10054496; doi:10.3390/jpm13030559)
Supplement: Supplementary file 1 [file jpm-13-00559-s001.zip › jpm-2246010-supplementary Table S1.pdf]

No	BSA	Saturation PA	Saturation Aorta	RA - A wave	RA - V wave	RA mean	RV Systolic	RVEDP	PA Systolic	PA Diastolic	PA mean	CPW A wave	CPW V wave	CPW mean	Cardiac Output Thermo	Cardiac Index Thermo	Cardiac Output Fick	Cardiac Index Fick	PVR	SVR	PVR	SVR
1	1.78				14	10	90	11	68	23	43		22	17	3.1	1.72	5.9	3.28	5.2		2.7	
25	1.94	78	94	16	16	13	37	12	37	19	25	19	23	18	4.6	2.42	8.3	4.37	1.6	17.6	0.9	9.7
52	2.30	77	96	8	6	6	31	16	28	18	24	15	14	13	6.1	2.8	10.3	4.6	1.8	16.4	1.1	11.6
3	2.49	74	96						65	26	35	25	25	20	3.6	1.50	6	2.50	4.2		2.5	
58	1.49	78.4	96.7	10	8	7	38	10	31	10	20	13	15	12	3.9	2.6	5.9	4	2.0	21.7	1.3	14.1
15	1.52	69	95	13	10	9	37	13	41	9	22	17	20	12	2.8	1.87	4.21	2.81	3.6	38.3	2.3	25.0
54	1.97	56	96	12	9	7	84	12	86	34	50	25	23	15	4.6	2.4	6.6	3.4	7.6	20.5	5.3	14.2
13	2.29	76	95	4	3	1	41	10	31	12	20	13	12	9	6	2.73	8.6	3.91	1.8	13.3	1.2	9.2
9	1.61	72		9	9	7	50	11	45	16	29	16	27	17	3.7	2.31	5.3	3.31	3.3		2.3	
19	2.01	76	100	15	17	13	82	18	86	43	58	38	37	37	4.7	2.47	6.7	3.53	4.5		3.1	16.3
43	1.90	82	98	6	3	2	37	10	33	13	22	12	14	10	5.5	2.9	7.7	4	2.2	21.0	1.6	5.1
12	2.08	72.7	91	12	12	8	48	9	38	14	26	25	29	21	5.7	2.85	7.7	3.85	2.3	22.9	1.7	16.8
14	1.87	80.8	95	13	4	4	37	4	37	11	22	13	14	10	5.7	3.17	7.7	4.28	2.1		1.6	13.0
32	1.82	80	98	3	1	-1	37	3	36	9	18	10	9	6	5.2	2.89	6.9	3.83	1.5		1.2	
56	1.76	76	91	9	7	6	35	11	40	14	25	19	16	14	6	3.5	7.8	4.5	1.5	13.4	1.2	10.4
65	2.03	79	96	8	7	5	33	10	27	11	18	17	16	10	6.8	3.4	8.8	4.4	1.2	15.8	0.9	12.1
59	1.94	57	97	11	15	10	41	11	51	20	35	16	28	24	2.9	1.5	3.6	1.9	3.9		3.1	18.5
17	1.93	80	96	8	8	7	51	11	52	21	34	20	14	12	6.9	3.63	8.5	4.47	3.1	11.0	2.6	8.9

10	1.75	73.7	98	10	8	7	33	14	26	5	15	13	10	9	4.2	2.47	5	2.94	1.3		1.1	16.7
44	2.02	71	98	10	10	7	35	11	31	17	22	24	24	20	3.8	2	4.5	2.3	0.4	26.5	0.4	22.5
18	2.11	76.5	95				36	10	33	17	24	18	20	13	6.6	3.30	7.8	3.90	1.8		1.5	
23	1.67	79	97	7	8	5	28	10	20	9	14	13	16	10	6.1	3.59	7.2	4.24	0.6	12.0	0.5	10.0
37	1.94	78	98	17	9	8	28	11	32	11	17	14	17	13	5.2	2.70	6.1	3.21	0.8	21.5	0.7	14.5
5	2.43	56	88	32	33	26	100	28	108	41	67	26	37	28	5.8	2.52	6.8	2.96	6.7		5.7	
49	1.89	62	85						45	26	35	14	13	12	6.6	3.6	7.6	4.2	3.4		3.0	
48	2.28	68	93	16	16	15	48	17	19	18	17	26	32	26	5.3	2.4	6.1	2.8	0.9		0.8	
61	1.65	70	95	15	13	10	34	17	34	12	22	18	28	16	4	2.4	4.6	2.9	1.4	20.0	1.2	17.1
22	1.97	77	95	5	4	3	27	7	23	9	15	12	12	10	6.4	3.37	7.1	3.74	0.8	16.0	0.7	14.4
21	1.79	64	94.6	8	5	5	44	13	43	24	31	17	20	16	3.8	2.11	4.2	2.33	3.8	40.5	3.2	36.9
45	1.94	70	96	24	16	17	45	23	42	28	35	42	28	35	6	3.2	6.55	3.4	1.5	13.8	1.4	12.7
68	1.34	58	90		26	21	76	26	81	38	58		48	35	3.7	2.7	4	2.9	8.0	36.4	7.5	33.9
64	2.09	67	95	15	16	13	52	18	52	27	38	25	23	22	5	2.6	5.4	2.8	3.2	23.5	3.0	21.9
51	1.31	76.4	97.3	3	4	1	48	7	44	9	24	15	13	9	6.2	4.6	6.54	4.9	2.4	21.2	2.3	20.0
2	1.62	42	92	23	24	21	81	26	86	48	59	23	23	22	2.3	1.44	2.4	1.50	11.3	38.9	15.4	
16	1.59	80	100	9	7	6	39	16	26	15	19	15	16	12	5.5	3.44	5.7	3.56	1.5	21.0	1.4	20.5
66	1.80	53	88	10	8	7	88	11	90	34	54	12	12	10	3.4	2	3.5	2	12.9	20.1	11.6	19.7
6	1.65	63	92	5	3	2	41	9	43	16	25	13	13	11	5.1	3.00	5.2	3.06	2.8		2.8	
4	1.59	77	100	5	4	1	28	8	29	13	21	13	17	10	5.1	3.19	5.2	3.25	2.1		2.1	
20	2.02	68.4	97	9	7	7	39	12	39	13	24	13	12	11	5.3	2.65	5.4	2.70	2.5	15.6	2.4	
60	1.97	76	97	16	15	12	43	19	34	19	26	21	25	19	5.5	2.9	5.6	2.9	1.3	18.3	1.3	18.3

67	1.46	75.7	98.7	10	9	8	47	12	26	12	18	19	29	15	4.84	3.4	4.85	3.41	0.7	17.0	0.7	17.0
26	1.46	59	98	18	15	15	47	21	46	21	33	28	32	23	4.2	2.10	4.2	2.10	2.3	20.9	2.2	20.9
11	1.63	79	98						28	12	19	13	17	10	6.1	3.81	6.1	3.81	1.5	13.8	1.5	13.9
7	1.53			4	2	1	25	6	23	5	11	10	11	6	4.9	3.27	4.9	3.27	1.4		1.4	
27	1.95	69	96	5	3	0	29	8	25	5	14	10	8	9	5.2	2.74	5.19	2.73	1.7	17.1	1.8	17.8
36	1.89	61	97		21	14	62	11	60	35	42		26	24	4.2	2.30	4.1	2.20	4.2	13.0	4.4	
8	1.84	76	94	9	8	4	42	12	44	19	31	16	16	13	8	4.44	7.4	4.11	2.2	8.6	2.4	9.2
24	2.05	48	90	22	21	19	111	27	106	37	60	11	10	9	4.9	2.45	4.5	2.25	10.4	14.6	11.4	15.9
31	1.53	68	95	21	11	9	37	16	34	17	23	23	19	18	3.5	2.33	3.2	2.13	1.6	35.7	1.7	38.5
33	1.48	46	76	18	18	16	104	16	109	31	59	16	19	15	3.8	2.53	3.4	2.27	11.8	22.3	12.9	
42	2.27	56	92	24	28	22	86	32	90	37	52	25	22	24	5.3	2.4	4.6<br>5	2.11	5.3		3.4	
35	1.77	76	97	7	6	4	32	12	26	9	16	16	19	12	5.8	3.41	4.5	2.65	0.7	25.3	0.5	15.9
30	1.47	78	100	10	18	10	59	14	55	27	37	22	27	20	4.5	3.00	3.3	2.20	3.8	26.3	5.2	35.8
29	1.94	72	98	15	16	13	43	14	40	18	29	21	34	22	4.9	2.58	3.3	1.74	1.4	21.3	2.0	31.9
28	1.57	66	98	3	5	1	49	6	55	25	38	30	34	26	3.8	2.57	2.5	1.69	3.2	32.4	4.6	49.2
